# Supplementary material for: Identifying degradation patterns of lithium ion batteries from impedance spectroscopy using machine learning
Source: Nat Commun. 2020 Apr 6;11:1706. doi: 10.1038/s41467-020-15235-7 (PMC7136228; doi:10.1038/s41467-020-15235-7)
Supplement: Supplementary file 2 — Supplementary Information [file 41467_2020_15235_MOESM2_ESM.pdf]

Supplementary Information

Identifying degradation patterns of lithium ion batteries from impedance spectroscopy using machine learning

Zhang et al.

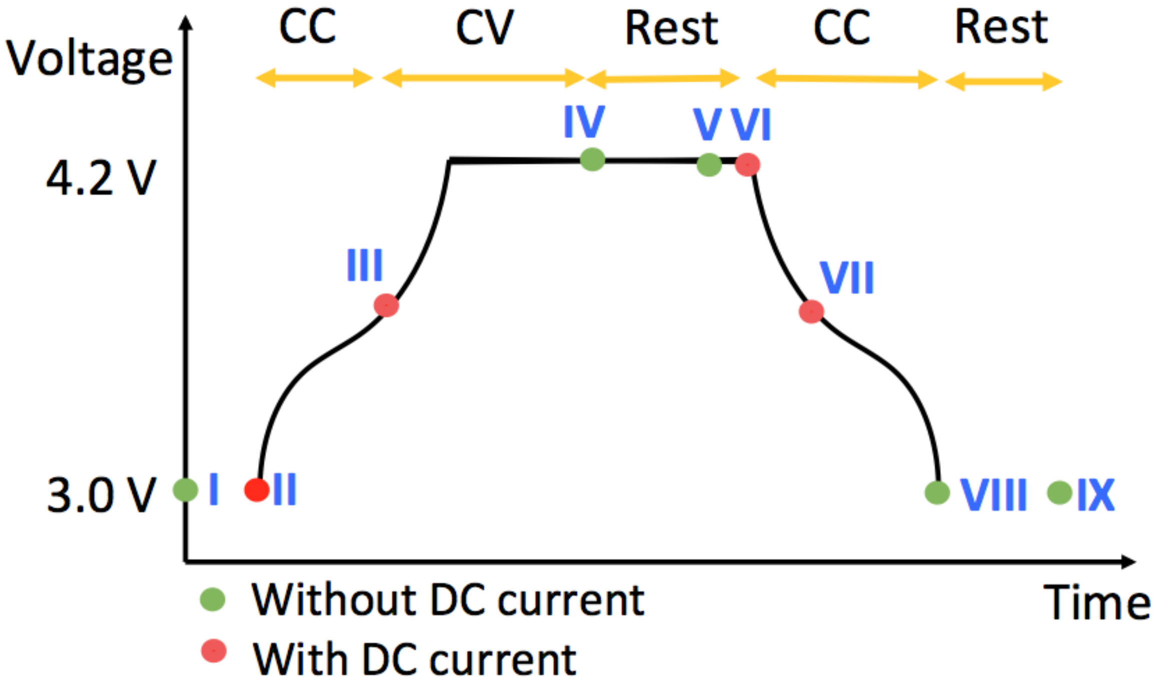

Supplementary Figure 1 – EIS data are collected at nine different states (representing as I-IX) during constant current-constant voltage (CC-CV) charging and discharging: I: Before charging; II: Start charging; III: After 20 minutes charging; IV: After charging and before resting; V: After 15 minutes rest; VI: Start discharging; VII: After 10 minutes discharging; VIII: After discharging and before resting; IX: After 15 minutes rest. The red (green) dots correspond to with (without) DC current.

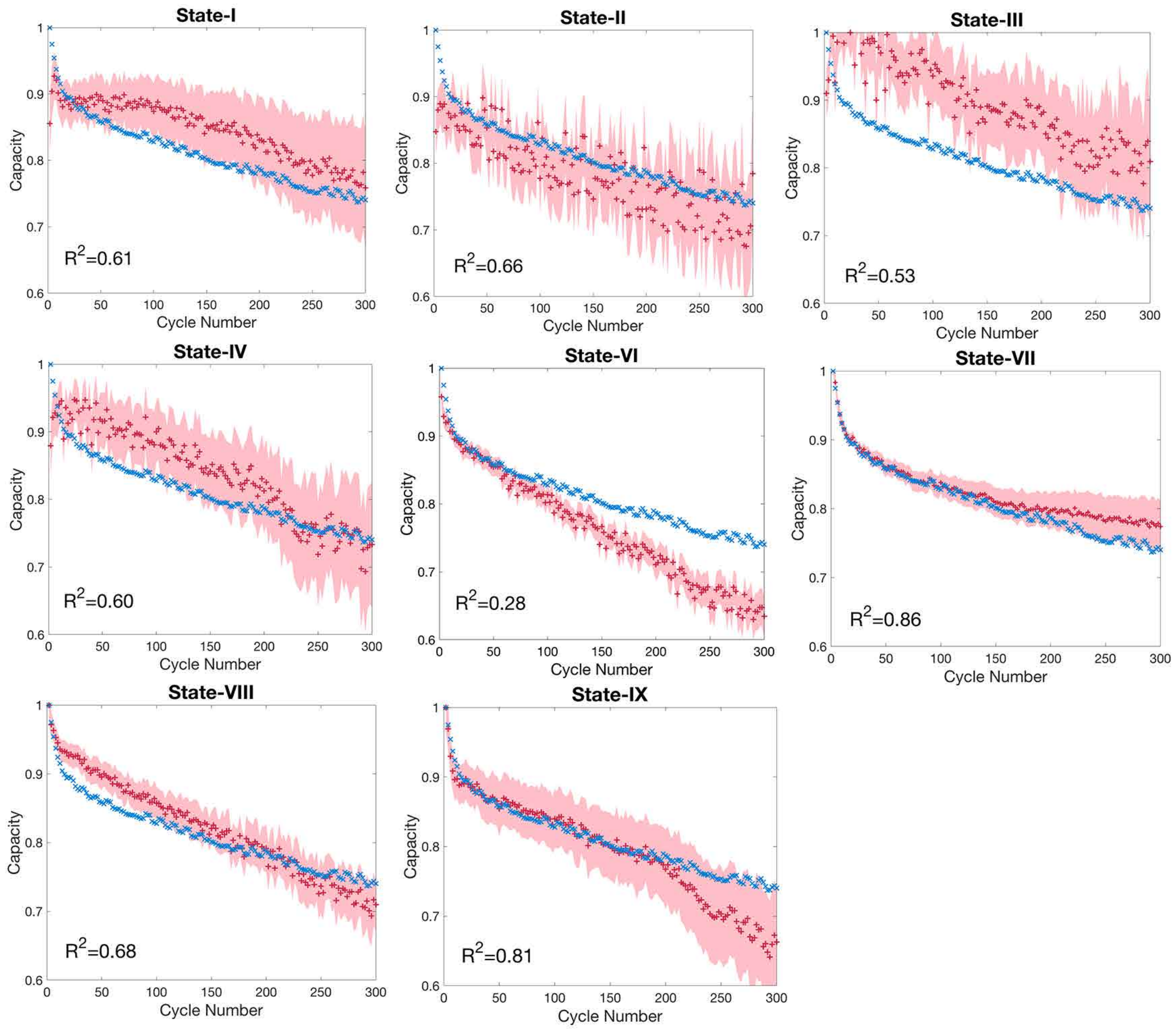

Supplementary Figure 2 – The estimation results of the EIS-Capacity GPR model for 25C02 cell cycled at the other eight different states (I-IX). The red curve shows the estimation of capacity, and the shaded region indicates $\pm$ 1 standard deviation. The testing EIS in each panel are collected at states I-IX, respectively. The blue dashed lines are the measured capacity. The coefficient of determination (R^2) is shown on the left bottom in each panel.

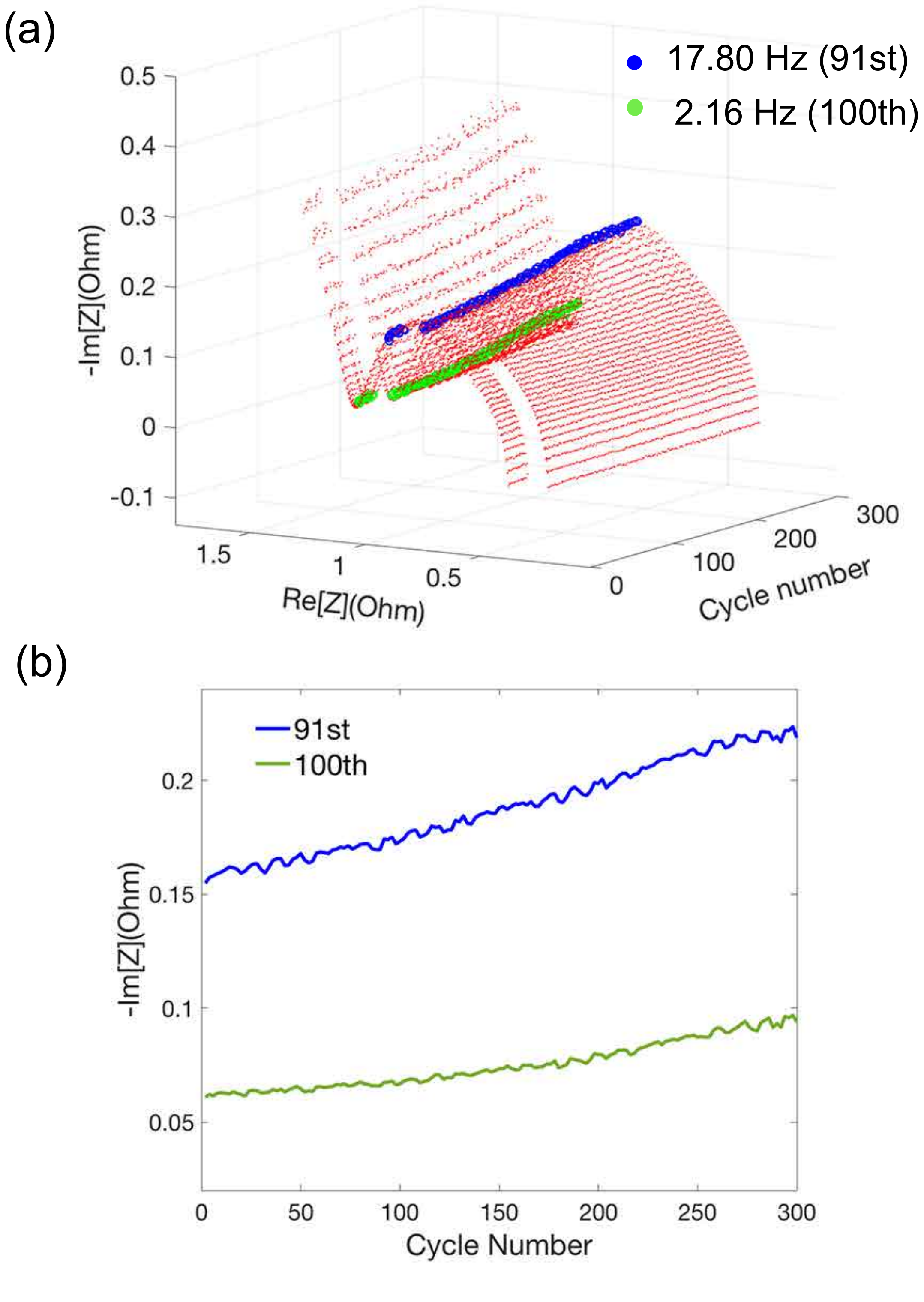

Supplementary Figure 3 – (a) The EIS spectra mapping along cycle numbers of 25C01 cell at state V (red dots). Blue and green curves indicate the two most salient frequencies of 17.80 and 2.16 Hz, respectively. (b) The imaginary part of the salient frequencies show a positive linear correlation with the cycle number.

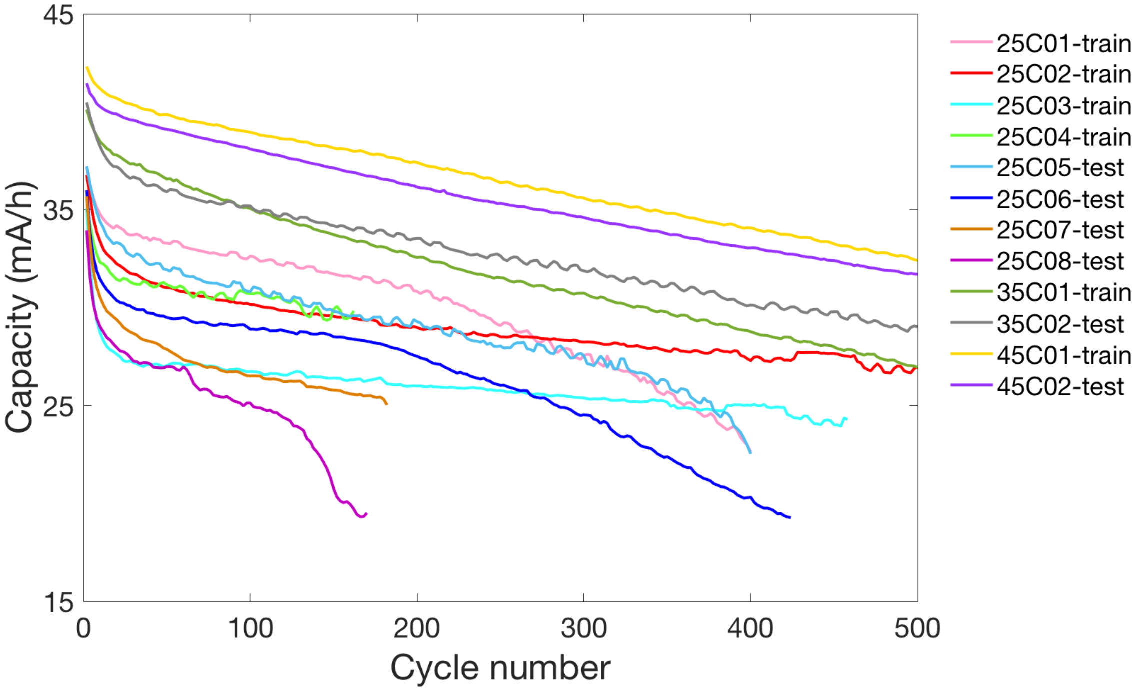

Supplementary Figure 4 – The capacity retention curves of all cells (marked with different colors). 25C01-25C04, 35C01 and 45C01 cells are training group. 25C05-25C08, 35C02 and 45C02 are testing group.

Input \ Cells	25C05	25C06	25C07	25C08
EIS (ours)	8.57	18.19	5.25	5.03
Capacity and voltage curves	43.22	34.28	38.14	73.20

Supplementary Table 1. The root-mean-squared error (RMSE) of our EIS-based method and the method using the discharging curve as input.
